# Extraction of Projectiles Lodged in the Hilum of the Lungs

**Published:** 1918-10

**Authors:** 


***Posterior Thoraco-Pneumotomia, Radio-Operatory, for the Extraction of Projectiles Lodged in the Hilum of the Lungs.*** By PETIT DE LA VILLÉON. *Bulletin de la Société de Chirurgie*, June 4, 1918. Presented by J. L. FAURE.

Dr. Petit de la Villéon presented sometime ago five or six wounded, perfectly cured, on whom he had operated four projectiles in the hilum. He had, at that time, operated on sixteen men with 100 per cent. of success.

These are very fine results, considering all the gravity of projectiles in the hilum, and Faure thinks it would be profitable to make known the study of the anatomical research which Petit de la Villéon went through in order to arrive at such results.

Petit de la Villéon defines the dangerous hilum region of the lungs as follows: anomo-vertebral trapezium limited by the spine, by the spinal edge of the scapula in its lower half, and by the fifth and eighth rib. His more detailed researches lead him to say that the most direct trans-pulmonary way of access to the hilum is the posterior. This method permits, instead of cutting deeply into the flesh, the resection of a rib, either the sixth, seventh or eighth, as the case may be. The scapula is not resected, only slightly deplaced at its lower angle by making the patient put his hand over his head. There is, therefore, much less danger of pleural infection. Petit de la Villéon calls attention to the fact that, if the projectile is located and seized with the X-ray light, the actual extraction is performed in daylight where the doctor can cope with any possible hemorrhage or other secondary accidents, and do the plugging in absolute safety. This plugging is removed forty-eight hours later, and too much care cannot be taken then to avoid hemorrhage. There was not a single one out of the sixteen cases referred to above.

J. L. Faure is convinced that the radioscopic is the best method of extraction. For surface projectiles the electro-vibrator is an admirable apparatus if the projectile is magnetic. For many projectiles localizing compasses give splendid results, but of course radioscopy gives the most certain results. It has its defects, the greatest of which is not the danger incurred by the surgeon, but rather the necessity of a very special and complicated equipment, and also the re-education of the eye, sometimes impossible for older men. Mauclaire even thinks it is not unimportant to bring up before doctors the fact that they must be patient in all radioscopic examinations, for it takes quite ten minutes for the retina to adapt itself and for the projectile to be seen at all.

Le Fort, who has often presented men operated on for intrathoracic projectiles, prefers the anterior extraction; that is to say, large transpleural extirpation. If radioscopy is not possible, undoubtedly this method is the safest, but it means, among other dangers, cutting deeply into the tissues. Lefort himself recognizes that the four unsuccessful cases he had out of a series of sixty operations were precisely those operated on the pulmonary pedicule. That is why the author thinks that if radioscopy were used, as it should be when circumstances allow, the posterior way is by far preferable.

Robert Didier advocates another method of resection which bears on costo-transversary articulation instead of making a gap by resecting one of the ribs. But the author thinks this little gap is not so injurious since it does not provoke abundant bleeding, is not deep nor difficult, and does not impair the strength of the thoracic framework.

J. L. Faure suggests that projectiles situated in front of the hilum should be reached by the chest, while those situated in the posterior region should be reached by the back so as to meet as few vessels as possible.

